# Assessment of the pathogenicity of the p.K695R and p.A744S Mediterranean fever gene variants

**DOI:** 10.1186/1546-0096-13-S1-P122

**Published:** 2015-09-28

**Authors:** Y Shinar, E Giat, R Cohen, A Livneh

**Affiliations:** 1 Sheba Medical Center, Heller Institute of Medical Research, Ramant Gan, Israel

## Question

How strong is the association of the NM_000243:c.2230G>, p. Ala744Ser variant and the c.2084A>G, p.Lys695Arg, with an FMF phenotype? The worldwide population frequency of these variants is 0.001-0.005, and they are uncommon among Israeli referrals for genetic diagnosis of FMF.

## Methods

Pathogenicity is indicated by the degree of positive association between the variant in its hetero- and monozygous forms, and an FMF phenotype. Subjects genotyped with p.K695R or p.A744S from 1500 referrals for FMF genetic testing between 2010-14 performed at the Sheba Medical Center, were clinically characterized using medical records or phone interview, by an expert blinded to the genotype. FMF diagnosis, and severity assessed by the Mor score were related to the genotype.

## Results

The p.K695R variant was found in 10 referrals (0.003 frequency) one of whom had a second, p. E148Q variant. All but 2 patients with IBD comorbidity had mild autoinflammatory symptoms. FMF criteria were met in one patient with IBD genotyped p.[R695K(;)E148Q] and 3 heterozygotes (40%). A North African Jewish or Arab ancestry was associated to this variant. The p.A744S variant was found in 17 referrals (0.006 frequency) 7 of whom, 5 heterozygotes and two compound heterozygotes met FMF clinical criteria (41%) with mild severity. However, a homozygous subject and two sibs with a second, p.M694V clearly pathogenic variant were not diagnosed with FMF. p.A744S referrals had in common a Mediterranean ancestry.

## Conclusion

The high frequency of FMF among patients carrying the p.A744SS and the p.K695R variants favors the inclusion of these variants within the spectrum of mild pathogenic mutations.

